# Supplementary material for: Modeling glioblastoma heterogeneity as a dynamic network of cell states
Source: Mol Syst Biol. 2021 Sep 16;17(9):e10105. doi: 10.15252/msb.202010105 (PMC8444284; doi:10.15252/msb.202010105)
Supplement: Supplementary file 6 — Source Data for Figure 5 [file MSB-17-e10105-s004.zip › Figure5A_sourcedata/GSEA_3071/nosigdb_stateVI.GseaPreranked.1622795169056/SINGH_SOX2_DOWNREGULATED_TRANSCRIPTS_PMID_28122245.html]

Details for gene set SINGH\_SOX2\_DOWNREGULATED\_TRANSCRIPTS\_PMID\_28122245[GSEA]

|  || Dataset | state63071 |
| Phenotype | NoPhenotypeAvailable |
| Upregulated in class | na\_pos |
| GeneSet | SINGH\_SOX2\_DOWNREGULATED\_TRANSCRIPTS\_PMID\_28122245 |
| Enrichment Score (ES) | 0.32285884 |
| Normalized Enrichment Score (NES) | 2.0974767 |
| Nominal p-value | 0.0 |
| FDR q-value | 0.0036789265 |
| FWER p-Value | 0.024 |
Table: GSEA Results Summary

  

Fig 1: Enrichment plot: SINGH\_SOX2\_DOWNREGULATED\_TRANSCRIPTS\_PMID\_28122245      
 Profile of the Running ES Score & Positions of GeneSet Members on the Rank Ordered List

  

| PROBE | GENE SYMBOL | GENE\_TITLE | RANK IN GENE LIST | RANK METRIC SCORE | RUNNING ES | CORE ENRICHMENT || 1 | SFTA1P |  |  | 4 | 1.263 | 0.0382 | Yes |
| 2 | MFAP5 |  |  | 22 | 1.009 | 0.0649 | Yes |
| 3 | LOX |  |  | 29 | 0.968 | 0.0933 | Yes |
| 4 | GATA3 |  |  | 36 | 0.929 | 0.1206 | Yes |
| 5 | KRT8 |  |  | 42 | 0.895 | 0.1471 | Yes |
| 6 | KCNMA1 |  |  | 55 | 0.861 | 0.1705 | Yes |
| 7 | PTX3 |  |  | 56 | 0.860 | 0.1973 | Yes |
| 8 | ANXA2 |  |  | 79 | 0.783 | 0.2155 | Yes |
| 9 | TAGLN |  |  | 100 | 0.737 | 0.2329 | Yes |
| 10 | DLC1 |  |  | 129 | 0.696 | 0.2467 | Yes |
| 11 | NABP1 |  |  | 186 | 0.609 | 0.2500 | Yes |
| 12 | ARHGAP22 |  |  | 193 | 0.607 | 0.2672 | Yes |
| 13 | RAB34 |  |  | 220 | 0.589 | 0.2783 | Yes |
| 14 | TACC1 |  |  | 245 | 0.568 | 0.2893 | Yes |
| 15 | SYNJ2 |  |  | 270 | 0.555 | 0.2998 | Yes |
| 16 | PLK2 |  |  | 363 | 0.505 | 0.2898 | Yes |
| 17 | CYR61 |  |  | 377 | 0.498 | 0.3017 | Yes |
| 18 | PLAGL1 |  |  | 400 | 0.490 | 0.3108 | Yes |
| 19 | TICAM2 |  |  | 412 | 0.485 | 0.3229 | Yes |
| 20 | RAB11FIP1 |  |  | 706 | 0.395 | 0.2533 | No |
| 21 | UBASH3B |  |  | 820 | 0.373 | 0.2334 | No |
| 22 | TRAF1 |  |  | 1040 | 0.339 | 0.1827 | No |
| 23 | RPS27L |  |  | 1136 | 0.325 | 0.1663 | No |
| 24 | COMMD1 |  |  | 1145 | 0.324 | 0.1742 | No |
| 25 | LURAP1L |  |  | 1149 | 0.323 | 0.1834 | No |
| 26 | RPS27A |  |  | 1194 | 0.317 | 0.1810 | No |
| 27 | IL15 |  |  | 1353 | 0.297 | 0.1461 | No |
| 28 | PLEKHG3 |  |  | 1412 | 0.291 | 0.1389 | No |
| 29 | RDH14 |  |  | 1437 | 0.286 | 0.1411 | No |
| 30 | DSE |  |  | 1484 | 0.282 | 0.1370 | No |
| 31 | MAN2A1 |  |  | 1507 | 0.279 | 0.1396 | No |
| 32 | PSMD7 |  |  | 1518 | 0.277 | 0.1454 | No |
| 33 | ECHDC1 |  |  | 1561 | 0.273 | 0.1422 | No |
| 34 | KRT18 |  |  | 1570 | 0.272 | 0.1484 | No |
| 35 | ACSL1 |  |  | 1605 | 0.268 | 0.1473 | No |
| 36 | PRICKLE1 |  |  | 1678 | 0.262 | 0.1353 | No |
| 37 | HS3ST5 |  |  | 1703 | 0.260 | 0.1367 | No |
| 38 | DPCD |  |  | 1719 | 0.259 | 0.1406 | No |
| 39 | SFT2D1 |  |  | 1721 | 0.258 | 0.1483 | No |
| 40 | TBXAS1 |  |  | 1779 | 0.253 | 0.1403 | No |
| 41 | TSPYL1 |  |  | 1935 | -0.269 | 0.1054 | No |
| 42 | SVIL |  |  | 1939 | -0.269 | 0.1129 | No |
| 43 | CPVL |  |  | 2000 | -0.278 | 0.1048 | No |
| 44 | CREM |  |  | 2019 | -0.282 | 0.1086 | No |
| 45 | DIXDC1 |  |  | 2043 | -0.284 | 0.1110 | No |
| 46 | FAM107B |  |  | 2234 | -0.320 | 0.0678 | No |
| 47 | PGBD5 |  |  | 2456 | -0.368 | 0.0176 | No |
| 48 | SERTAD4 |  |  | 2523 | -0.383 | 0.0111 | No |
| 49 | MAF |  |  | 2633 | -0.408 | -0.0067 | No |
| 50 | UNC5C |  |  | 2672 | -0.417 | -0.0043 | No |
| 51 | OMD |  |  | 2721 | -0.431 | -0.0043 | No |
| 52 | IGF2R |  |  | 2838 | -0.469 | -0.0221 | No |
| 53 | DCN |  |  | 3027 | -0.554 | -0.0574 | No |
| 54 | GSN |  |  | 3041 | -0.559 | -0.0436 | No |
| 55 | CXCR4 |  |  | 3302 | -0.759 | -0.0926 | No |
| 56 | HIST1H1C |  |  | 3509 | -1.145 | -0.1145 | No |
| 57 | TMEM132A |  |  | 3555 | -1.383 | -0.0840 | No |
| 58 | IGFBP3 |  |  | 3588 | -1.623 | -0.0424 | No |
| 59 | COL1A2 |  |  | 3602 | -1.803 | 0.0101 | No |
Table: GSEA details [plain text format]

  

Fig 2: SINGH\_SOX2\_DOWNREGULATED\_TRANSCRIPTS\_PMID\_28122245: Random ES distribution      
 Gene set null distribution of ES for **SINGH\_SOX2\_DOWNREGULATED\_TRANSCRIPTS\_PMID\_28122245**
